# Post-translational modifications in DNA topoisomerase 2α highlight the role of a eukaryote-specific residue in the ATPase domain

**DOI:** 10.1038/s41598-018-27606-8

**Published:** 2018-06-18

**Authors:** Claire Bedez, Christophe Lotz, Claire Batisse, Arnaud Vanden Broeck, Roland H. Stote, Eduardo Howard, Karine Pradeau-Aubreton, Marc Ruff, Valérie Lamour

**Affiliations:** 1 Université de Strasbourg, CNRS UMR7104, INSERM U1258, Institut de Génétique et de Biologie Moléculaire et Cellulaire, Illkirch, France; 2 Hôpitaux Universitaires de Strasbourg, Strasbourg, France; 3 Instituto de Física de Líquidos y Sistemas Biológicos, Conicet, La Plata, Argentina

## Abstract

Type 2 DNA topoisomerases (Top2) are critical components of key protein complexes involved in DNA replication, chromosome condensation and segregation, as well as gene transcription. The Top2 were found to be the main targets of anticancer agents, leading to intensive efforts to understand their functional and physiological role as well as their molecular structure. Post-translational modifications have been reported to influence Top2 enzyme activities in particular those of the mammalian Top2α isoform. In this study, we identified phosphorylation, and for the first time, acetylation sites in the human Top2α isoform produced in eukaryotic expression systems. Structural analysis revealed that acetylation sites are clustered on the catalytic domains of the homodimer while phosphorylation sites are located in the C-terminal domain responsible for nuclear localization. Biochemical analysis of the eukaryotic-specific K168 residue in the ATPase domain shows that acetylation affects a key position regulating ATP hydrolysis through the modulation of dimerization. Our findings suggest that acetylation of specific sites involved in the allosteric regulation of human Top2 may provide a mechanism for modulation of its catalytic activity.

## Introduction

Type 2 DNA topoisomerases (Top2) are part of essential protein complexes involved in DNA replication, chromosome condensation and segregation, as well as gene transcription^1–3^. The Top2 are highly conserved enzymes and catalyze the transport of a DNA duplex through a transient double strand break to release catenates, relax supercoils and remove entanglements^4^.

**Figure 1:**
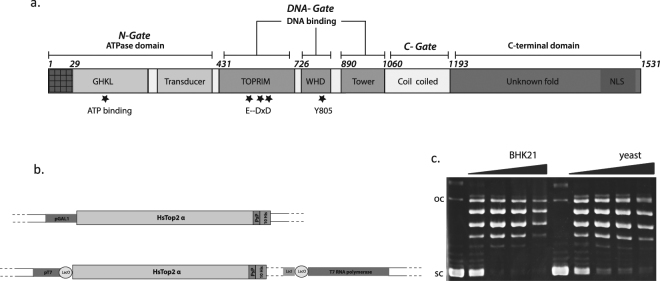
Production of the Top2α enzyme in yeast and mammalian cells. (**a**) Domain organization of the human Top2α. The ATPase domain contains 2 subdomains, the “GHKL” ATP binding domain and the transducer domain forming the N-Gate. The DNA binding site comprises 3 subdomains and form the DNA-gate: the Toprim domain containing the acidic triad E–DXD involved in metal binding, the Winged Helix Domain (WHD) with the conserved catalytic tyrosine, and the tower domain. The C-gate is formed by the coiled coil domain followed by the C-terminal domain containing Nuclear Localization Signal (NLS). (**b**) Overexpression constructs in yeast and mammalian cells. The Top2α gene was cloned after the pGal1 promoter for expression in yeast. For expression in BHK21 mammalian cells, the Top2α gene is integrated in the modified virus Ankara (MVA) genome. The LacO operator is upstream of T7 RNA pol and the Top2α genes, allowing a double control of both Top2α and T7 RNA polymerase expression by LacI. PsP: PreScission protease cleavage site, 10 His: 10 histidines tag. (**c**). DNA relaxation assay of the recombinant produced in yeast and mammalian cells. Negatively supercoiled DNA was incubated with increasing concentration of Top2α (0, 8, 17, 36, 72 nM) and migrated in a 1% agarose gel incubated with ethidium bromide. oc: open circular DNA, sc: supercoiled DNA.

Top2 are among the main targets of anticancer agents, prompting intensive efforts to understand the functional and physiological role of this enzyme family as well as their molecular structure^5^. Vertebrate organisms possess two isoforms, Top2α and Top2β, encoded by distinct genes^6–8^. The human Top2 isoforms share considerable sequence identity and form functional homodimers with three protein interfaces or gates comprising the ATPase domain, the DNA binding-cleavage domain and the C-terminal domain (Fig. 1a). Tridimensional structures of the ATPase and the DNA binding domains have been solved by X-ray crystallography^9–11^. The α and β isoforms diverge mainly in their C-terminal domain, whose structure remains unknown and may account for the functional differences of the isoforms. Expression of the Top2α isoform is regulated in a cell cycle dependent manner in proliferative cells^12^. The β isoform plays a more specialized physiological role as this isoform has been shown to be essential for the final stage of neural differentiation^13,14^.

Post-translational modifications of Top2 isoforms have been found to influence enzyme activity, stability and subcellular localization^15^. Among them, phosphorylation sites are the best characterized with a number of studies showing their impact on the regulation of Top2 enzyme function, nuclear localization and control of cell cycle checkpoint^16–22^. Both isoforms of human Top2 are phosphorylated throughout the cell cycle and some positions are hyperphosphorylated during G2/M^21,23–25^. In addition, ubiquitination, SUMOylation and several acetylation events were identified but the correlation with the cell cycle, protein recruitment or regulation of Top2 function is not yet fully understood^26,27,15^. Furthermore, inhibition of Top2α by anti-cancer drugs is known to be modulated by phosphorylation^28–30^. Hyperphosphorylation of the α isoform has been reported in etoposide resistant cells, while other studies associated a hypophosphorylation of the enzyme after doxorubicin treatment^29,31,32^.

Overexpression protocols in the yeast system were set up for the yeast enzyme in the late 80 s, and for the human isoforms in the early 90 s^33–35^. Despite improvements of protocols to obtain pure protein, eukaryotic systems used to generate recombinant Top2 are expected to produce a complex set of posttranslational modifications that remains largely uncharacterized and could impact *in vitro* functional and inhibition assays. Further studies are important to characterize such modifications.

In this work, we have mapped and compared phosphorylation and acetylation sites of the human Top2α isoform overexpressed in *S*. *cerevisiae* and BHK21 mammalian cells. We report the acetylation sites of the Human Top2α isoform (HsTop2α), clustered on the catalytic domains. Using point mutation, biochemical assays, biophysics approaches and molecular dynamic simulations, we have analyzed the role of a eukaryotic-specific position located in the ATP binding pocket of the enzyme, highlighting the catalytic mechanism that is targeted by acetylation.

Overall, our study shows that post-translational modifications may impact conserved residues of Top2α and not only variable regions It also suggests that the post-translational modifications of the recombinant enzymes should be taken into account when analyzing their functional properties and sensitivity to inhibitors.

## Results

### Optimization of the HsTop2 expression in yeast and mammalian cells

cDNA for the Top2 isoforms were inserted in a pYEP24 derived yeast expression plasmid using restriction enzymes more than two decades ago^34,35^. To facilitate the purification of these large enzymes, we added a 10-histidine tag and a protease cleavage site at the 3′ end of the genes (Fig. 1b). The plasmids were transformed in the yeast JEL1 Gal^+^ strains auxotrophic for uracil as previously described^35^. To improve expression, we switched from a lactate/glycerol medium to a raffinose medium that is not repressive, allowing a faster induction of expression^36^. Overall, we could shorten and simplify the expression protocol to 3 days from plasmid transformation to cell harvesting compared with former published procedures. The 170 kDa protein was purified using a nickel affinity and then a heparin chromatographic step with a final yield of 1 mg per liter of culture (Figure S1).

The overexpression of the enzyme in BHK21 mammalian cells was achieved using the Modified vaccinia virus ANkara (MVA) encoding the full length genes as a vector under the control of a T7 promoter (Fig. 1b). This strategy was previously used to successfully express eukaryotic proteins^37^. The protein purification was performed with the protocol optimized for the HsTop2α overexpressed in *S*. *cerevisiae*. The final yield obtained from MVA infected BHK21 cells was 1.5 mg per liter of culture (Figure S1).

### Characterization of the *in vitro* activities of the recombinant Top2α isoform

We compared the catalytic activities of the recombinant protein produced from *S*. *cerevisiae* (Sc-HsTop2a) and BHK21 cells (BHK21-HsTop2a). DNA relaxation assays using the recombinant Sc-HsTop2a and BHK21-HsTop2a show that the enzyme starts relaxing negatively supercoiled DNA with a protein concentration of 8 nM and that the DNA is fully relaxed at 17 nM for both enzymes (Fig. 1c). These data are in agreement with published data indicating that the recombinant enzymes are similar and fully functional for DNA relaxation activities when produced from either of the two expression systems^34^.

We also determined the ATP hydrolysis rate of Sc-HsTop2a and BHK21-HsTop2a with a steady state coupled PK/LDH ATPase assay^38^. Both enzymes produced in yeast or mammalian cells have an equivalent rate of ATP hydrolysis with a k_cat_ of 2.05 ± 0.035 s^−1^ for Sc-HsTop2a and 2.7 ± 0.5 s^−1^ for BHK21-HsTop2a. These values are similar to the values found for the endogenous and overexpressed enzymes previously reported in the literature^39,40^.

### Identification of the HsTop2a phosphorylation and acetylation sites

**Figure 2:**
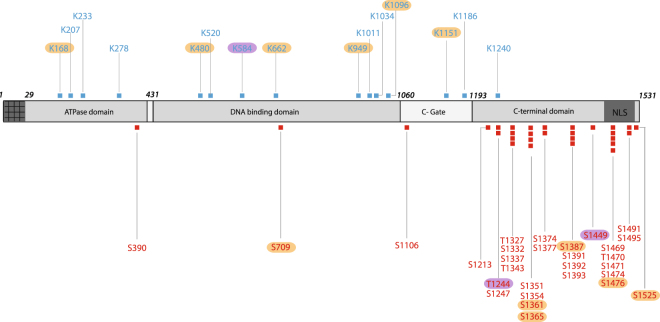
Post-translational modifications of the human Top2α identified by mass spectrometry. Phosphorylation (red squares) and acetylation (blue squares) sites are reported on the HsTop2α primary sequence. Modifications that were mostly detected in Sc-HsTop2a or in BHK21-HsTop2a in the experimental replicates are highlighted in orange and in magenta respectively.

The purified recombinant enzymes were trypsin-digested and the peptides analyzed by LC-MSMS using an Orbitrap mass spectrometer. Phosphorylated peptides were enriched through affinity columns prior to analysis. Overall, 86% of the peptide sequence was covered following the in-gel trypsin digestion. To compensate for the relative abundance of particular modified positions, we performed at least 3 separate experiments to complete the analysis (supplemental material 5). We detected 27 phosphorylation sites and 14 acetylation sites for the Sc-HsTop2a protein, and 23 phosphorylation sites and 9 acetylation sites for the BHK21-HsTop2a protein (Fig. 2). In the technical replicates, 21 phosphorylation and 8 acetylation sites were consistently found at the same positions in the recombinant protein monomer expressed in yeast or mammalian cells, suggesting an equivalent level of modification in both eukaryotic expression systems. Using a malachite green assay^41^, we were able to calculate that the Top2α samples produced in yeast or BHK21 cells contain an average of 7 ± 1 phosphates per Top2α subunit, meaning that at most one third of all sites are phosphorylated. There is no available standard deacetylation assay that we could use to assess the acetylation status of our sample. Similar to the heterogeneity in phosphorylation, we expect that the Top2α sample produced in yeast and mammalian cells is only partially acetylated.

**Figure 3:**
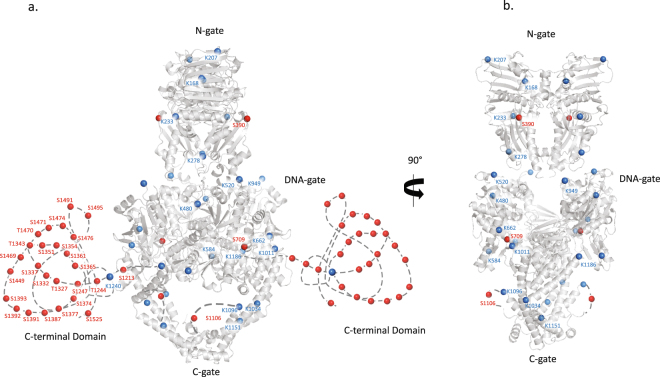
Distribution of acetylation and phosphorylation sites on the Top2α structural domains. Identified phosphorylation (red) and acetylation (blue) sites were reported on the human Top2α domain structures. The ATPase domain (PDBID: 1ZXM) and the DNA binding domain without DNA (PDBID: 5GWK) of the Top2α isoform appear in grey cartoon. Position numbers have been inserted only on one of the monomer chain of the dimeric structure for clarity. (**a**) Front view of the structure with the C-terminal domains (CTD) of the eukaryotic Top2 depicted as a dashed line on each side of the catalytic core. (**b**) Side view of the structure showing the position of modifications on the catalytic domains in a different orientation (structures rotated 90° relative to a.) The CTD domains have been omitted for clarity. A movie is provided in the supplemental material. Figures were generated using PyMOL Molecular Graphics System, Version 2.0 Schrödinger, LLC.

Phosphorylated residues were mostly clustered in the C-terminal domain, with only 1 phosphorylation in each of the ATPase, DNA binding and C-gate domains. On the contrary, acetylation sites were found homogeneously on residues located in the ATPase and DNA binding domain, with only one acetylated residue was at the entrance of the C-terminal domain in a region that is not visible in the available crystal structures (Fig. 3). The acetylation sites are spread out over the catalytic domains with 10 positions on the DNA gate and C-gate, and 4 in the ATPase domain.

#### Functional analysis of position K168

**Figure 4:**
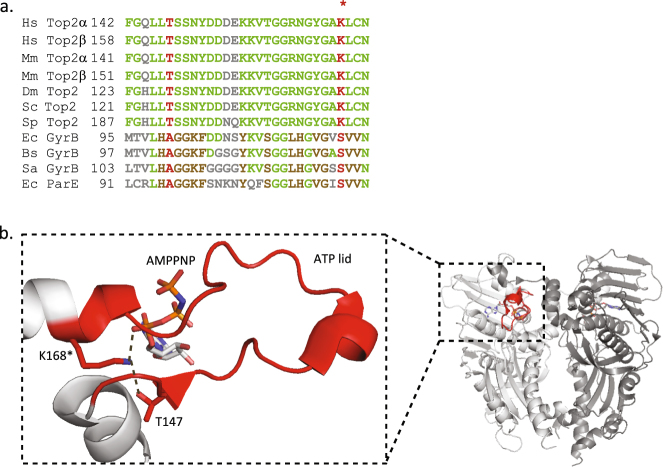
Interactions mediated by residue K168 in the ATPase domain. (**a**) Sequence alignement of the ATP lid. Conserved positions are colored in green, conserved positions in the prokaryotic sequences in brown. Positions delimiting the beginning and end of the loop are indicated in red. The acetylated position K168 in the HsTop2α is indicated by a star. *Hs: homo sapiens*, *Mm: mus musculus*, *Dm: drosophila megalonaster*, *Sc: saccharomyces cerevisiae*, *Sp: saccharomyces pombe*, *Ec: escherichia coli*, *Bs: bacillus subtilis*, *Sa: staphylococcus aureus*. b. Structure representation of the ATP lid. The ATPase domain of the human Top2α isoform (PDB:1ZXM) appears as a light gray ribbon with the ATPase domain loop (ATP lid) in red circling the γ-phosphate of the AMP-PNP molecule. K168 is located at the basis of the ATP lid and forms 2.8 Å hydrogen bonds with the AMP-PNP α-phosphate and Thr147 (dashed lines). Figures were generated using PyMOL Molecular Graphics System, Version 2.0 Schrödinger, LLC.

Among the ATPase domain positions, Lysine 168 was found acetylated only once out of several determinations in the recombinant Top2α sample produced in yeast, indicative of a low occurrence of this modification. Lysine 168 lies in a conserved sequence motif within the GHKL domain of eukaryotic Top2 and is replaced by a Serine in prokaryotic organisms (Fig. 4a). This residue is positioned at the base of the ATP lid, a flexible loop wrapping around the ATP molecule (Fig. 4b). As revealed by the sequence alignment, the primary sequence of the ATP lid is largely divergent between eukaryotes and prokaryotes. The interactions maintaining the base of the loop in presence of ATP are as a consequence driven by different interaction networks (Figs 4b and S5). Lysine 168 is forming a direct hydrogen bond with the ATP molecule. This led us to analyze more precisely the role of this position in the catalytic function of the human Top2α.

**Figure 5:**
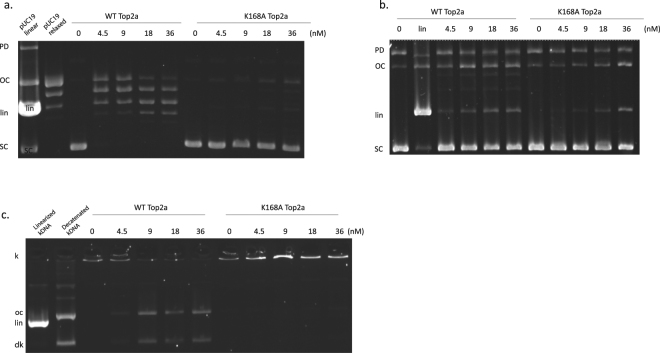
Activities of the Top2α-K168A mutant. (**a**) DNA relaxation assays showing a decrease in activity for the mutant enzyme. (**b**) DNA cleavage assays with etoposide show an impaired cleavage of the K168A mutant when compared with the same concentrations of WT enzyme. (**c**) Decatenation assays show that the mutant is inactive when compared with the WT enzyme. Reactions were run on a 1% agarose gel incubated with ethidium bromide. OC = open circular DNA, SC = supercoiled DNA, lin = linear DNA, PD = pUC19 dimers, k = catenated DNA, dk = decatenated DNA.

To probe the role of lysine 168, we mutated Lysine 168 to Alanine in the full length enzyme (HsTop2a^K168A^). The mutant was overexpressed in yeast and purified with our optimized protocol resulting in a similar yield as the WT enzyme. DNA relaxation and cleavage activities are impaired for the K168A mutant when compared with the same concentration range of the WT enzyme (Fig. 5a,b). The ATPase domain mutation has a strong impact on the decatenation activity, which is completely abolished (Fig. 5c). ATP hydrolysis enzymatic assays showed that catalytic activity of the mutant is decreased as much as 7-fold (K_cat_ = 0,3 ± 0.06 s^−1^) compared with the WT (K_cat_ = 2.05 ± 0.035 s^−1^) although not completely abolished.

**Figure 6:**
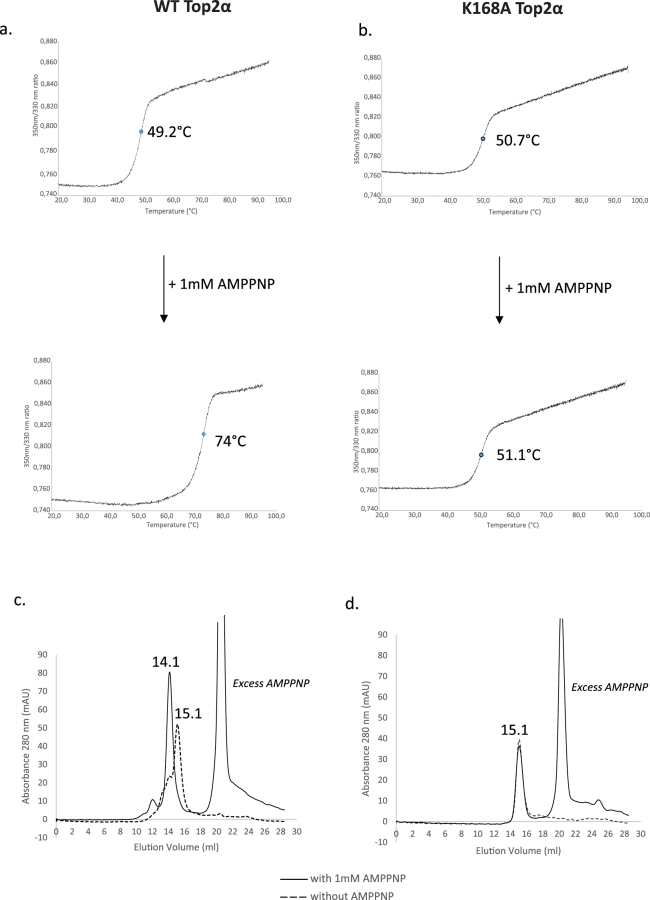
Thermal stability and dimerization analysis of the Top2α WT and K168A mutant. (**a**) The thermal shift of the domains was measured by differential scanning fluorimetry thermal shift assay. The fusion temperature of the wild type ATPase domain shifts from 49.2 °C to 74 °C in presence of AMPPNP (**b**) The mutant domain K168A presents about the same melting temperature of 51 °C in presence or absence of AMPPNP. (**c,d**) The wild type and mutant ATPase domain were analyzed by analytical gel filtration on a Superdex200 (10/300). The wild type domain (**c**) elutes at 15.1 ml alone and at 14.1 ml with 1 mM AMPPNP with a significant shift corresponding to the dimer formation. The K168A mutant (**d**) elutes at 15.1 ml with and without AMP-PNP consistent with no dimer formation. Elution volumes are reported to the first decimal for clarity, detailed analysis of the elution peaks is provided in Figure S5.

To probe the role of K168 in the stabilization of the ATPase domain, we purified the ATPase domain (residue 29 to 428) bearing the K168A mutation (ATPase^K168A^), as well as the WT domain, using a modified version of a previously published protocol^9^. To analyze the stability of the ATPase^K168A^ domain compared with the wild type ATPase domain, we performed thermal shift assays using differential scanning fluorimetry *(NanoDSF)*^42^. The wild type ATPase domain displays a melting temperature (Tm) of 49.2 °C with a thermal shift to 74 °C in presence of AMP-PNP, a non hydrolysable analog of ATP (Fig. 6a). The thermal shift of over 20 °C is consistent with a stabilization of the ATPase domain in the presence of AMP-PNP, in agreement with the well documented transition from the monomeric to the dimeric state of ATPase domains, upon AMP-PNP binding. Ionic strength variations in the buffer conditions did not affect the transition of the melting temperature, indicating a very strong stabilization of the WT domain in the presence of AMP-PNP (data not shown). For the mutant domain ATPase^K168A^, we recorded a Tm of 50.7 °C without AMP-PNP, and 51.1 °C when AMP-PNP was added (Fig. 6b). The temperature shift of 0.4 °C within the error margin suggests no stabilization of the ATPase^K168A^ domain in the presence of AMP-PNP.

To investigate the dimerization state of the mutant and wild type domains, we performed an analytical size exclusion chromatography with and without AMP-PNP. The ATPase domain was eluted at 15.1 ml with a shift to 14.1 ml in the presence of AMP-PNP (Fig. 6c). This 1 ml elution shift corresponds to the formation of a 94 kDa dimeric form in agreement with the calibration curve of the gel filtration column. The ATPase^K168A^ domain was eluted at 15.1 ml, both in absence and in presence of AMP-PNP (Fig. 6d), corresponding to the 47 kDa monomeric form of the ATPase domain, showing that no dimerization occurs for the mutant domain in the presence of AMP-PNP (Figure S6).

#### Molecular modeling and dynamic simulation

Normal mode analysis is a computational method to study molecular deformational motions that are widely involved in biological functions of macromolecules. It was shown in previous studies that the lowest-frequency modes contribute significantly to large-scale displacements and can provide important information on domain motions^43^. Focusing only on these modes, we quantified the intrinsic dynamics (flexibility) of the homodimer and the effects of both the K168A mutation, as well as K168 acetylation on the ATP binding domain monomer. The results for the atomic fluctuations of the wild-type dimer were compared with the fluctuations calculated from the crystallographic B-factors of the corresponding crystal structure (Figure S2). A good correspondence is found between the computed and experimental values. We find that loop regions, and to some degree, the N- and C-terminals of the protein display the highest flexibility. While this general dynamical behavior is also evident from the temperature factors, this overall favorable comparison establishes that our calculations provide a good representation of the molecular-level dynamics. To assess the impact of the point mutation K168A and the potential effect of K168 acetylation *in silico*, we investigated the changes in low-frequency vibrational modes on the monomer. The calculations show that the point mutation K168A or acetylation of K168 in the ATPase domain results in a change in the collective motions of the monomeric chain with respect to the wild-type, particularly affecting the N-terminal arm of the monomer (Figure S3). We expect similar consequences for the higher frequency vibrational modes. These results are suggestive of the effect of point mutations and post-translational modification on the underlying dynamics of the monomeric chain, which may subsequently have consequences for the formation of the active dimer.

## Discussion

In the present study, we were able to improve the production pipeline in *S*.*cerevisiae* for the human Top2 isoforms and establish a robust overexpression protocol in BHK21 mammalian cells yielding active recombinant enzymes. In order to characterize the post-translational modification state of our samples, we identified the phosphorylation and acetylation sites of the HsTop2α overexpressed in the two eukaryotic expression systems. Our data show that the position and number of phosphorylation and acetylation sites were similar upon overexpression in either of the two systems. The yeast expression system being the most convenient choice to produce eukaryotic Top2, this study suggests that *S*. *cerevisiae* may be as efficient as higher eukaryotes to produce a properly modified HsTop2, at least for the most predominant post-translational modifications. At this stage, it is not straightforward to quantify the preponderant modifications in the samples. However, the resulting samples display a basal catalytic activity with catalytic constants similar to those reported in the literature for endogenous and recombinant enzymes and constitute what can be considered as the wild type reference used in the field.

As expected from the literature, our analysis identified a large number of phosphorylated sites located on the C-terminal domain of HsTop2α (Figs 2 and 3). This domain does not directly participate in the catalysis of ATP hydrolysis or DNA cleavage activity but it was shown to modulate DNA cleavage and the recognition of supercoil handedness^44,45^. It is also involved in determining subcellular localization and protein-protein interactions^46–48^. Top2α has been found to be a major component of chromatin associated complexes whose composition varies during mitotic progression^49–51^. The phosphorylated clusters confer a negative charge to this region, which could potentially trigger interactions with basic patches of other proteins and modulate binding to DNA. In this context, *in vitro* validation of Top2 partners should take into account the phosphorylation state of the enzyme.

Our data corroborate a number of the phosphorylation sites in databases reporting phosphoproteome analysis of cancer cell lines or targeted biochemical studies (Phosphosite plus, UniprotKB). We identified 29 phosphorylation sites over the 74 reported modifications in the Phosphosite Plus database^27^ (Table S1), with the exception of one modification at position S709, that was not described in previous reports. This shows that the modifications introduced by the heterologous overexpression systems are consistent with the ones reported for the endogenous Top2. The recombinant enzyme is a complex sample of protein modified at different steps of the cell cycle and only a subset of sites was found to be systematically modified upon each analysis. This is consistent with studies showing that specific positions are cell cycle dependent, while others are phosphorylated during the entire cell cycle^15^.

On the other hand, not all reported phosphorylation sites were present in the recombinant protein. Studies have shown that the level of phosphorylation can be modified when cells are treated with anti-topoisomerase drugs, revealing a correlation between the regulation of the phosphorylation state of Top2 and the cytotoxic response in a living cell. One study has shown that the decrease of Top2α cleavage-complexes is correlated with hypophosphorylation^28^. As a consequence, the additional sites reported in the database could indicate that some sites are modified only in cancer cell lines or when exposed to drugs. Further studies are warranted to determine if phosphorylation events can be attributed to specific cancer cell lines or to cellular responses to anti-topoisomerase molecules. In this context, the phosphorylation state of the recombinant Top2α enzyme should also be taken into account when used for drug screening.

In contrast to what is known about phosphorylation sites, only a few acetylated positions in the Top2 proteins were previously identified through proteomic analysis of post-translational modifications in cell lines^26,27^. Despite several large scale proteome analyses of eukaryotic cells there is no reliable prediction and consensus motif for acetylation sites, pointing to the necessity for experimental detection^52^. Our mass spectrometry analysis of the recombinant Top2α shows that the acetylation sites are distributed on the conserved ATPase and DNA binding domains forming the catalytic core of the enzyme (Fig. 3). This result is consistent with previous observations that acetylations are often found in structured regions while phosphorylations are observed in divergent disordered domains^52^. These modifications are mostly located on the surface of the enzyme in accessible regions, including DNA binding regions that can be exposed through conformational remodeling during the catalytic cycle of the enzyme.

Introduction of modifications on these mostly conserved positions is likely to affect the catalytic activity of the enzyme. In particular, we identified an acetylation on the conserved Lysine 168 located in the ATP binding pocket and at the center of an interaction network at the basis of the ATP lid (Fig. 4a,b). The release of the γ phosphate of ATP after hydrolysis breaks this interaction and induces a reorganization of the dimeric interface at the base of the ATP lid. Mutation of this residue to Alanine inhibited the relaxation and the decatenation activity of the enzyme, as well as the dimerization of the ATPase domain, highlighting its pivotal role in the dimerization-ATPase coupling reaction. The mutant enzyme is deficient in DNA relaxation but still retains DNA cleavage activity showing it can bind a DNA segment but cannot transport another DNA segment. Normal mode analysis confirms that mutation to Alanine could affect dimerization by perturbations of the native collective motions. The same *in silico* analysis conducted on the ATPase domain with an acetylated Lysine 168 suggests that this modification would also affect the collective motions that could subsequently affect dimerization. It is interesting to note that this residue is replaced by a Serine in bacterial type 2 topoisomerases that makes a hydrogen bond network through a water molecule contacting the α-phosphate of the AMP-PNP molecule (Figs 4 and S4). A lysine at this position in the human enzyme forms a much tighter interaction network and its acetylation would be a very efficient way to directly regulate the topoisomerase activities, in particular DNA capture that is correlated to dimerization events. Acetylation sites were also found in the DNA-binding cleavage domain along the DNA groove and in the C-gate region (Fig. 3 and supplemental movie). These modifications are most likely to affect DNA binding and transport and the resulting allosteric control of the protein gates.

No specific study has been conducted to understand the role of these modifications *in vivo* or *in vitro* although it is known that histone deacetylase 1 and 2 associate with the Top2 and enhance its activities^53^. The recognition of acetylated lysine is principally mediated by bromodomains within multidomain proteins which are well characterized for binding histone modifications in chromatin^54^. The Top2α proteins are part of large multiprotein complexes associated with chromatin remodeling. In particular, interaction with the SWI/SNF complexes containing bromodomains were reported to increase the Top2α decatenation and ATP hydrolysis activities^55^. Despite these recent findings, the molecular mechanisms regulating the acetylation/deacetylation level of Top2 has yet to be addressed.

It is noteworthy that acetylation of Lysine 168 was detected only once over seven analysis. As this residue is critical for enzyme activity, any modification could potentially represent an event of protein inactivation. Interestingly this residue was found to be ubiquitinated in proteomic analysis of leukemia cells^26^. 15 acetylation sites were found during our analysis and 7 of them were reported in the literature as ubiquitinylated and 2 as sumoylated sites in the PhosphositePlus databank. Although we did not detect the presence of SUMO or ubiquitin peptides in our analysis, it is interesting to note that a crosstalk between acetylations and sumoylation/ubiquitination has been reported on chromatin proteins^56^. Sumoylation of Top2 has been reported to prepare the enzyme for degradation by the 26 S proteasome and regulate cell response to drug-induced DNA damage^57,58^. In addition the SUMO2/3 proteins and SUMO ligases RanBP2 and PIASγ and have been shown to affect the localization of theTop2 at the centromere^15^. The tumor suppressor BRCA1 was shown to reduce Top2α activity through its E3 ubiquitin ligase activity^15^. Acetylation at key catalytic positions could potentially be part of regulating a signaling cascade shutting down activity before degradation of the Top2. The molecular interaction orchestrating the fine regulation of the multiple modifications of Top2 still need to be investigated. Our results open perspectives for the systematic analysis of the impact of acetylation on the catalytic and cellular activities of human Top2α. This will require detailed *in vitro* studies and determination of the Top2 functions in the cellular context.

## Methods

### *S. cerevisiae* expression plasmid

The Top2α gene was amplified by PCR from the *Saccharomyces* *cerevisiae* expression plasmid pWOB6-HsTop2 using a C-terminal primer containing a PreScission Protease (PsP) cleavage sequence followed by a 10-histidine tag. In this construct, the first five residues of S. cerevisiae Top2α are fused to the 29th residue of HsTop2a. PCR inserts were ligated in the yeast expression plasmid pYES2.1, a high-copy episomal vector for galactose-inducible expression of proteins in *Saccharomyces cerevisiae* (pYES2.1 TOPO TA Expression Kit, Invitrogen). Sequencing of the plasmid confirmed the insertion of the Top2 gene without any mutation.

### *S. cerevisiae* overexpression

For expression, we used the *Saccharomyces* *cerevisiae* strain, Jel1ΔTop1 (Mat α leu2 trp1 ura3-52 prb1-122 pep4-3 Δhis3::PGAL10-GAL4 Top1::ura3Δ). Yeast transformation was performed using the LiAc procedure^59^. Several colonies of freshly transformed Jel1ΔTop1 were used to inoculate 4 × 0.5 L of minimum media lacking uracil (Csm-ura), and supplemented with 2% (w/v) glucose to repress the GAL1 promoter. These pre-cultures were grown for 2 days at 30 °C. At the late log phase, cultures were pelleted at 3500 g for 20 minutes and the media containing glucose was thrown away. Pellets were then resuspended in 5 ml of Csm-ura media. 6 liters of Csm-ura, supplemented with 2% (w/v) raffinose and 2% (w/v) galactose medium were inoculated at a final OD of 0.8 per liter, and incubated for 16 hours at 30 °C. Due to the presence of galactose in the media, induction starts right away after inoculation. Finally, cells were pelleted at 3500 g for 20 minutes and flash-frozen in liquid nitrogen before storage at −80 °C until use. The overexpression of the Top2α isoform was confirmed by western blot with antibodies raised against the C-terminal domain of the enzyme.

### Construction of the BHK21-MVA expression plasmid

The full length HsTop2α gene, with the PsP cleavage site followed by a 10-histidine tag sequence, was amplified with primers containing attL1 and attL2 sites for Gateway cloning. These amplified sequences were introduced into the pENTR backbone (pE607 Invitrogen). The hsTop2α gene was then transferred by Gateway recombination in a pVote0GW vector containing a selective marker and a portion of the viral hemagglutinin gene sequence (HA)^60^. Generation of the recombinant viruses was performed as described previously^37^. Briefly, the plasmid resulting from the recombination between pE-E607-HsTop2α and pVote0GW was transfected into MVA-T7 infected BHK21 cells. During the virus replication, the HsTop2 coding sequence flanked by HA sites is recombined in the virus genome thanks to the HA sites. The resulting recombinant viruses are selected and amplified by an iterative selection process.

### BHK21 overexpression

For protein production, a suspension of BHK21 C13–2P cells (10^6^ cells/ml) was infected with approximately 0.1 PFU/cell of recombinant virus in 5 liter cylindrical flasks containing 1.2 liters cell culture medium (GMEM, 10% FCS, 1.5 g/L BTP, 50 μM Gentamycin). Two days later, infected cells were mixed with uninfected cells at a 1:10 ratio in six 5-liters cylindrical flasks containing 2 liters of uninfected cells at 10^6^ cells/ml. 1 mM IPTG was added at the time of cell mixture. Incubation at 37 °C was continued for another 24 hours. Cells were then pelleted at 2000 g for 5 minutes, washed in PBS and pelleted again at 2000 g for 5 minutes. The cell pellets were stored at −80 °C until use.

### Full length HsTop2 WT and K168A purification

The point mutation at position K168 was introduced in the pYES2.1-hsTop2α plasmid using the Quick Change kit (Invitrogen) for expression in yeast. All steps were carried out at 4 °C. The purification procedure was the same for *S*. *cerevisiae* and BHK21 cells. Cell pellets were resuspended with a 1:3 (w/v) ratio in a lysis buffer containing 50 mM HEPES pH8.0, 1 M NaCl, 10% glycerol, 0.1 mM MgCl2, 2 mM β-mercaptoethanol (BME), 1 mM PMSF and Complete protease inhibitor EDTA-free (PIC, Roche). Cell lysis was performed with high pressure Homogenizer Emulsiflex C3 by 4 to 5 passes at >1500 bars. Cell debris were removed by centrifugation at 50 000 g for 60 minutes. Briefly, the proteins were purified using two chromatographic steps using Nickel affinity and Heparin columns (see supplemental procedures for detailed procedures).

### HsTop2 WT and K168A ATPase domain production

The WT or K168A mutant ATPase domain sequence was amplified by PCR from the corresponding pYES2.1 vectors and inserted using a Ligation Independent cloning protocol in a pET28b-derived vector with an N-terminal 6-histidine tag, a Maltose Binding Protein fusion (MBP) and a TEV protease site. The vectors were transformed in a BL21 (DE3) strain and grown in 6 liters of LB medium in presence of 100 µg/l of Kanamycin at 37 °C. Expression was induced with IPTG at OD_600nm_ = 0.8 at 30 °C for 10 h. Cells were harvested and lysed 3 times with an Emulsiflex C3 homogenizer at 1500 bars in a buffer containing 50 mM Tris-HCl pH8.0, 500 mM NaCl, 5 mM MgCl_2_, 0.1 mM PMSF and protease inhibitor cocktail. Briefly, the proteins were purified using three chromatographic steps using Nickel affinity, MBP affinity and Heparin columns (see supplemental procedures for detailed procedures).

### DNA relaxation and cleavage assays

An increasing concentration of Top2α was incubated with 11 nmoles of negatively supercoiled pUC19 plasmid at 30 °C in a reaction mixture (20 µl) containing 20 mM Tris-acetate pH7.9, 50 mM potassium acetate, 10 mM magnesium acetate, 1 mM DTT, 1 mM ATP, 50 µg/ml BSA. After 20 minutes, reactions were stopped by addition of DNA loading buffer (Thermofisher) and SDS 0.2%. For cleavage assays, 50 µM of etoposide were added to the reaction and incubated 30 min at 37 °C. The reactions were stopped with 0.2% SDS and treated with 0.5 mg/ml proteinase K for 40 min at 37 °C. Samples were run on a 1% agarose, 1× Tris Borate EDTA buffer (TBE) gel, at 9 V/cm for 180 min at room temperature. Agarose gels were stained with 0.5 mg/ml ethidium bromide (EtBr) in 1 × TBE for 15 min, followed by 5 min destaining in water. DNA topoisomers were revealed using a Typhoon 8600 or Syngen U:Genius3 scanner.

### Decatenation assays

Decatenation assays were performed using 0.2 µg of kDNA per reaction (Topoisomerase II Assay kit from Topogen) with purified Top2α and incubated 30 min at 37 °C and run on a 1% agarose, 1× Tris Borate EDTA buffer (TBE) gel, at 5.5 V/cm for 1 h at room temperature. The reactions were stopped with 1% SDS and treated with 0.5 mg/ml proteinase K for 15 min at 37 °C and analyzed in the same conditions as the relaxation assays.

### Analytical gel filtration

The oligomerization state of the K168A mutant was analyzed by gel filtration chromatography with a Superdex200 10/300 GL column (GE Healthcare). Protein samples concentrated at 1.0 mg/ml with and without an excess of AM-PNP were injected on the column equilibrated against 20 mM Tris-HCl pH 8.0, 5 mM MgCl_2_, 200 mM NaCl and 0.1 mM BME, at a flow rate of 0.3 ml/min.

### Thermal shift assays

The thermal shift assay was performed on a Prometheus NT.48 apparatus (NanoTemper Technologies). This technique records the absorption of tryptophan and tyrosine at respectively 330 and 350 nm by Differential Scanning Fluorimetry (DSF) as the protein unfolds in a temperature gradient from 20 to 95 °C. The buffer solution and protein concentration were the same as the ones used for gel filtration.

### ATPase enzymatic assays

ATPase assays were performed as described by Lindsley *et al*.^61^. ATP hydrolysis is coupled with the oxidation of NADH through a cascade of pyruvate kinase (PK) and lactate deshydrogenase (LDH) reactions. The absorbance is monitored at 340 nm over 200 seconds at 37 °C with a Shimadzu 1700 spectrophotometer. Reactions were recorded in triplicates with 75 nM protein and 21 nM plasmid DNA (PUC19) in 500 µl of a buffer containing 50 mM Tris HCl pH7.5, 150 mM potassium acetate, 8 mM magnesium acetate, 7 mM BME, 100 µg/mg of BSA, 4U/5U of PK/LDH mixture, 2 mM PEP, and 0.2 mM NADH.

### Mass spectrometry analysis

Purified protein samples (5–10 µg) were loaded on a SDS polyacrylamide gel and bands were excised from one-dimension gel lane and subjected to manual in-gel reduction, alkylation and digestion. The gel bands were reduced with 10 mM DTT for 1 h at 57 °C and alkylated for 45 min in the dark with 55 mM iodoacetamide. Each band was duplicated and digested with 600 ng of trypsin (Promega) overnight at 37 °C. The extracted peptide solutions were then dried using a SpeedVac centrifuge (5301 Concentrator Eppendorf). Detailed procedures for the NanoLC-nanoESI/MS-MS analysis of the resulting peptides with an OrbiTrap ELITE mass spectrometer are provided in supplemental materials.

### Malachite green assay

Phosphate concentration was assessed using the Malachite Green Assay kit from Cayman chemical. 10 µg of HsTop2α was incubated for 30 minutes at 37 °C with increasing ratio of purified lambda phosphatase to release all phosphates from the recombinant protein. The reaction was performed in a buffer containing 50 mM Hepes pH 8.0, 2 mM DTT and 0.01% Brij 35. The reaction mix was then incubated with the malachite kit reagent and absorbances at 620 nm were measured in a 96 well plate on a Synergy HTX spectrophotometer. Experiments were run in triplicates. Phosphate concentration was assessed using the calibration curve and standard solutions provided in the assay kit.

### Molecular modeling

The structure of the ATPase domain of human DNA topoisomerase 2α dimer was obtained from the Protein Data Bank (PDBID: 1ZXM)^9^. For the modelling, the AMP-PNP was replaced by ATP in the binding pocket. The missing loops were constructed by homology modelling using the Modeller program^62^ and this structure was used for all subsequent calculations. All calculations were done using the CHARMM program^63^ and the all-atom force field for proteins^64^. Hydrogen atoms were placed using the HBUILD facility^65^ in the CHARMM program.The detailed procedures for normal mode analysis following a previously developed protocol^66^ are provided in supplemental materials.

All data generated or analysed during this study are included in this published article (and its Supplementary Information files).

## Electronic supplementary material

Supplemental information

Supplementary Dataset 1

Supplementary Dataset 2

Supplementary Movie
