# Publisher Correction: Post-translational modifications in DNA topoisomerase 2α highlight the role of a eukaryote-specific residue in the ATPase domain

**DOI:** 10.1038/s41598-018-28936-3

**Published:** 2018-07-10

**Authors:** Claire Bedez, Christophe Lotz, Claire Batisse, Arnaud Vanden Broeck, Roland H. Stote, Eduardo Howard, Karine Pradeau-Aubreton, Marc Ruff, Valérie Lamour

**Affiliations:** 1 Université de Strasbourg, CNRS UMR7104, INSERM U1258, Institut de Génétique et de Biologie Moléculaire et Cellulaire, Illkirch, France; 2 Hôpitaux Universitaires de Strasbourg, Strasbourg, France; 3 Instituto de Física de Líquidos y Sistemas Biológicos, Conicet, La Plata Argentina

Correction to: *Scientific Reports* 10.1038/s41598-018-27606-8, published online 18 June 2018

The original version of this Article was published with an incorrect version of the Supplementary Information file due to a technical error.

In addition, two Supplementary Datasets, and a Supplementary Movie showing distribution of acetylation and phosphorylation sites on the Top2α structural domains were omitted from the original version of this Article.

This has now been corrected in the HTML version of the Article; the PDF version of the paper was correct from the time of publication.

