# Supplementary material for: Post-translational modifications in DNA topoisomerase 2α highlight the role of a eukaryote-specific residue in the ATPase domain
Source: Sci Rep. 2018 Jun 18;8:9272. doi: 10.1038/s41598-018-27606-8 (PMC6006247; doi:10.1038/s41598-018-27606-8)

Supplementary Information

Post-translational modifications in DNA topoisomerase 2α highlight the role of a eukaryote-specific residue in the ATPase domain

Claire Bedez, Christophe Lotz, Claire Batisse, Arnaud Vanden Broeck, Roland H. Stote, Eduardo Howard, Karine Pradeau-Aubreton, Marc Ruff, Valerie Lamour

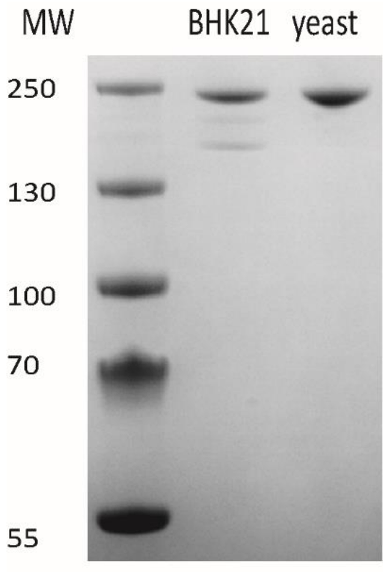

Figure S1. SDS-PAGE of the purified samples of HsTop2α produced in BHK21 mammalian cells and yeast. MW: molecular weight marker, gel colored by Coomassie blue.

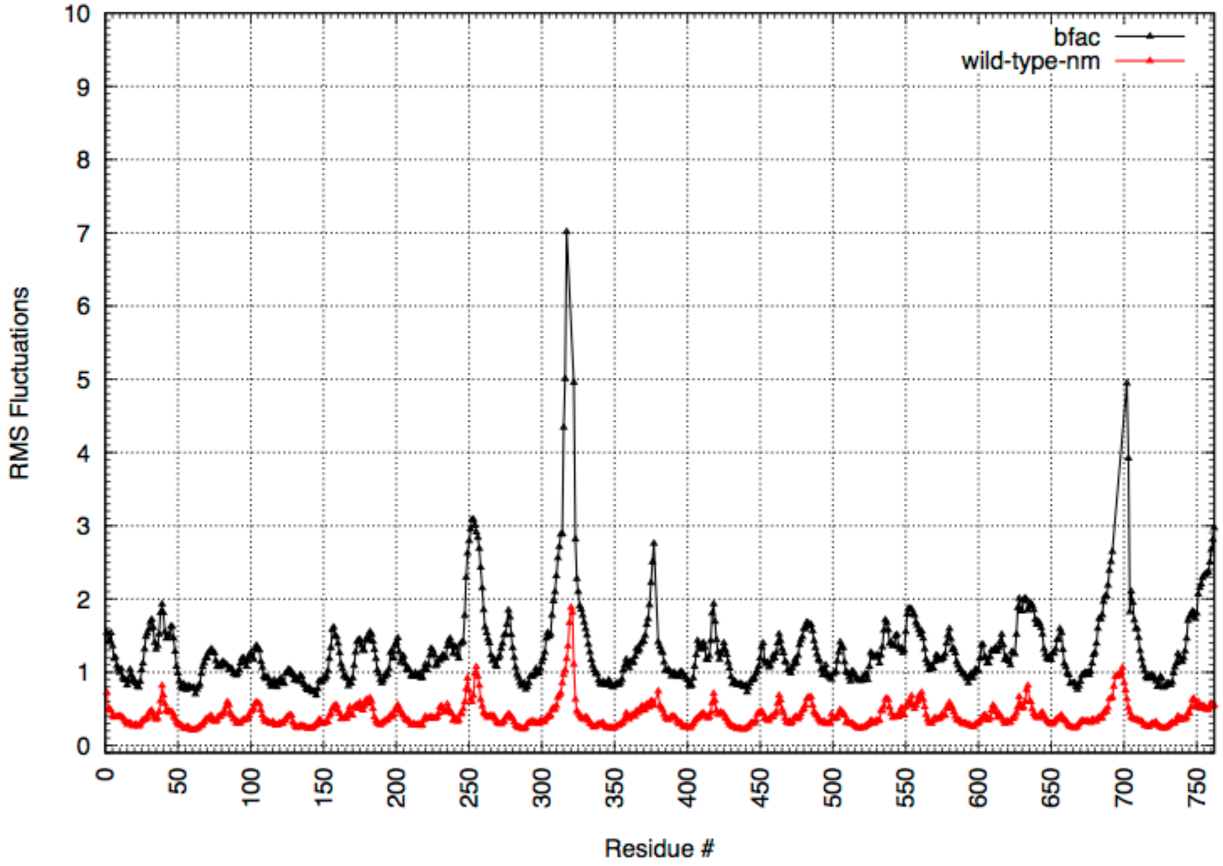

Figure S2. Atomic fluctuations in Å calculated from the normal modes (red) and experimental B-factors (black) for the wild type structure. The normal mode fluctuations are averaged over the 20 structures. The RMSF pattern obtained using the 500 lowest frequency vibrational modes corresponds well to that of the fluctuations calculated from the B-factors, although the fluctuations calculated from normal modes tend to be lower in absolute value than those derived from experimental ones. This is due, in part, to the contribution of crystal lattice disorder to the B-factors (62).

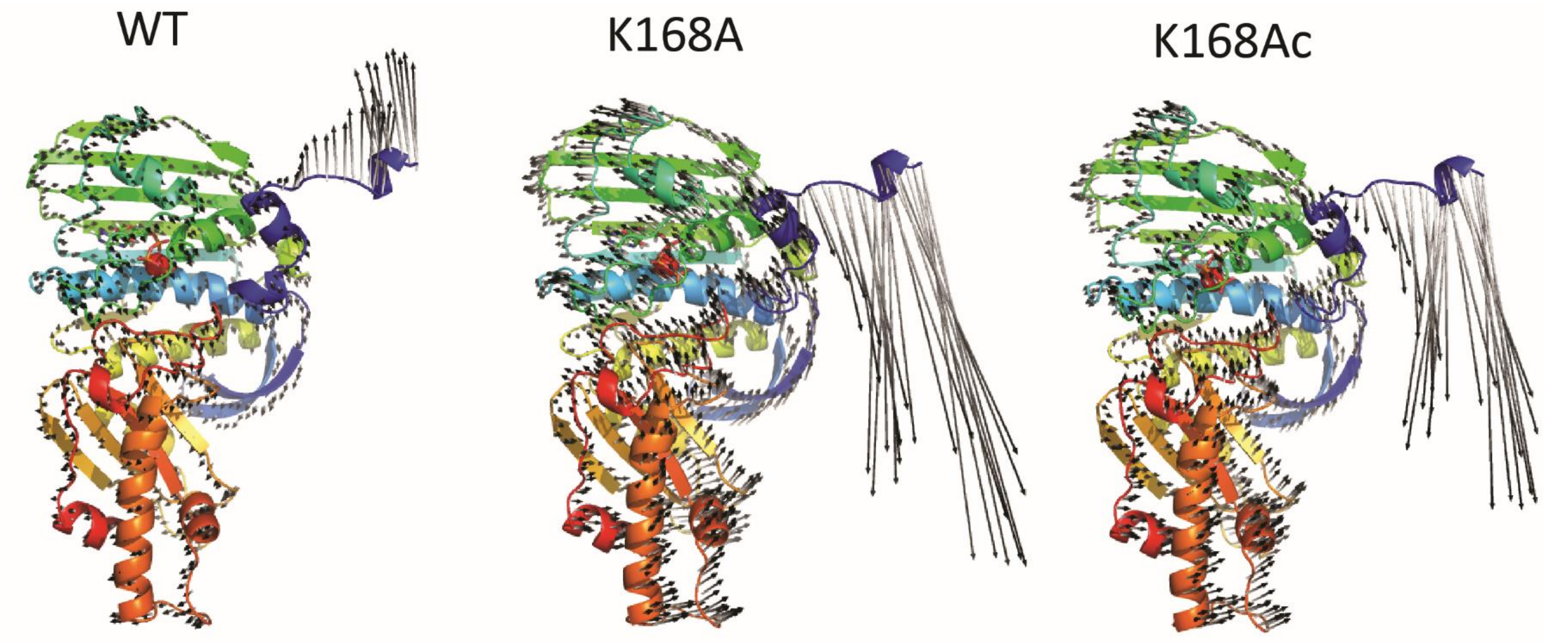

Figure S3. **Representation of the atomic displacements of the ATPAse domain monomer**. Backbone atomic components of the vibrational mode are represented as arrows and are associated to the lowest frequency vibrational mode of the monomeric chain averaged over the 20 calculations per form. Vector norms have been arbitrarily scaled and only vectors with the higher norms are represented for clarity. The spectrum color gradient is related to the sequence (N terminus is blue, C terminus is red), the metal ion is shown as a red sphere and the ATP as sticks. The atomic displacements are increased for the K168A mutant and for the K168Ac acetylated form in particular on the GHKL subdomain and the N-terminal arm suggesting that formation of the dimer would be affected.

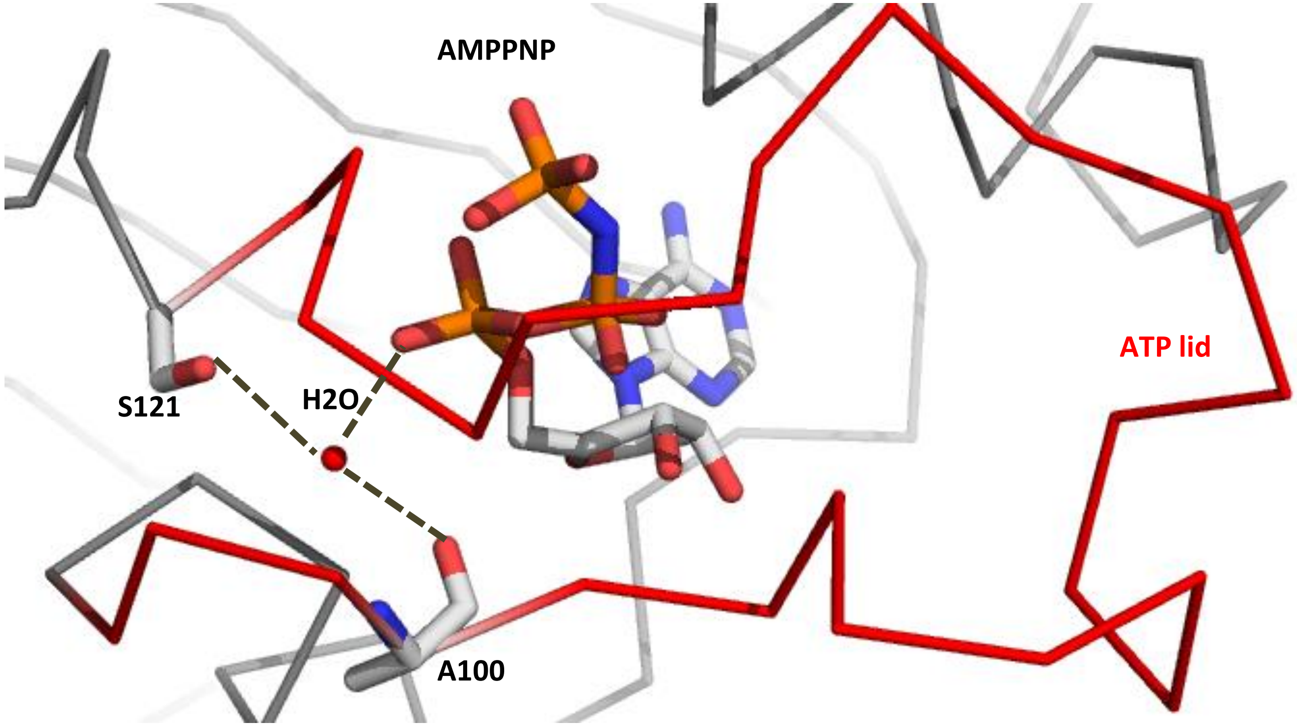

Figure S4. Interactions mediated by residue S121 in the ATPase domain of bacterial Top2 at the equivalent position of K168. The ATPase domain of the E. coli Gyrase B (PDB:1EI1) appears as a light gray ribbon with the ATPAse domain loop (ATP lid) in red circling the γ-phosphate of the AMPPNP molecule. S121 is replacing K168 in the bacterial sequence and is located at the basis of the ATP lid. This residue forms indirect hydrogen bonds through a water molecule with the AMPPNP α-phosphate and A100 (dashed lines). Figures were generated using PyMol Molecular Graphics System (Version 1.8 Schrödinger, LLC).

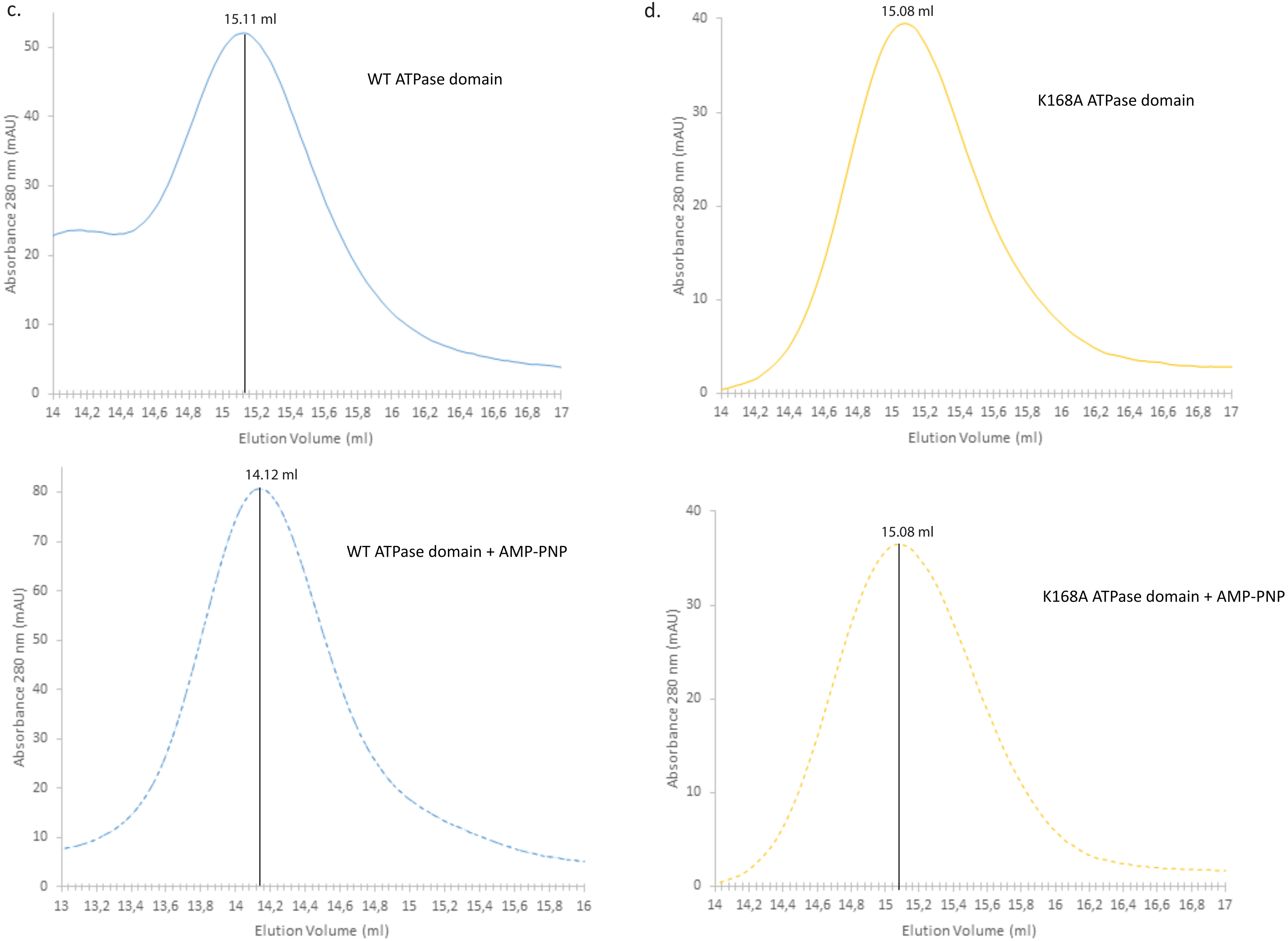

Figure S6: Magnified view of the elution peaks corresponding to the gel filtration chromatograms of Figure 6 c (WT ATPase domain) and d. (K168A ATPase domain). The center of the peak is indicated by a vertical line with the precise volume value.

Supplemental experimental procedures:

Full length HsTop2 WT and K168A purification (chromatographic steps):

The supernatant was loaded on a Nickel Hitrap Chelating HP 5ml (GE Healthcare) pre-equilibrated with lysis buffer. After three washing steps with 20 mM, 35mM and 60 mM imidazole, the Top2α protein was eluted by the addition of 250 mM imidazole in the lysis buffer. To decrease the NaCl salt concentration and remove imidazol, the eluted proteins were dialyzed overnight against 50 mM HEPES pH 8.0, 10% glycerol, 400 mM NaCl, 2mM BME, and with PsP at a ratio of 1:50 mg (PsP:Top2). The sample was then loaded on Heparin column (HiTrap Heparin 1ml GE Healthcare) pre-equilibrated with the dialysis buffer. Finally, the protein was eluted at about 800 mM NaCl, by a linear salt gradient. Purified protein aliquots were flash-frozen in liquid nitrogen and stored at -80°C until use.

HsTop2 WT and K168A ATPase domain purification:

After centrifugation at 20000g, the lysis supernatant was loaded on a Nickel column (HiTrap Chelating HP 5ml, GE Healthcare). The protein was first washed with 30 mM Imidazole and eluted with a buffer containing 50 mM Tris-HCl pH8.0, 150mM NaCl, 400mM Imidazole, 5mM $MgCl_2$, 2 mM BME, directly into a Heparin column (HiTrap Heparin HP 5ml, GE Healthcare) pre-equilibrated with 20 mM Tris-HCl pH8.0, 150 mM NaCl, 5 mM $MgCl_2$, 2 mM BME. The protein was eluted in a single step with a buffer containing 20mM Tris pH8.0, 5 mM $MgCl_2$, 450 mM NaCl, 2mM BME. The eluate was then loaded on an amylose column (MBP Hitrap HP 5 ml, GE Healthcare) and eluted with a buffer containing 10 mM Maltose. TEV protease was added to the eluate at a 1:30 ratio and incubated 12h at 4°C. The cleaved mixture was loaded on the Nickel column in tandem with the Heparin column equilibrated with 20 mM Tris-HCl pH8.0, 150mM NaCl, 5mM $MgCl_2$, 30 mM Imidazole 2mM BME. The 6-histidine-MBP remains bound to the Nickel column while the ATPase domain is eluted from the Heparin column at high salt concentration. Purified protein aliquots were flash-frozen in liquid nitrogen and stored at -80°C until use.

Mass spectrometry analysis:

Phosphopeptide enrichment: Samples were suspended in loading buffer [50% acetonitrile (ACN), 0.1% trifluoroacetic acid (TFA)], incubated with PHOS-Select iron affinity beads (Sigma Aldrich) at a ratio 1/2.5 (µL beads volume / µg peptide mixture) for 30 min and loaded into a 200 µL Gel-Loader tip (Costar). After washing with the binding and washing (20% ACN, 1% TFA) buffers, phosphopeptides were eluted with 4% ammonium hydroxide, desalted on Graphite spin-column (Pierce-Thermo Fisher Scientific) and dried (Speed-vacuum).

NanoLC-nanoESI/MS-MS analysis: Digested acetylated or phosphorylated peptides were resuspended in 5 µL of 0.1% HCOOH and between 3 or 5 µL were injected in the nano liquid chromatography in conjunction with collisional tandem mass spectrometry system (nanoLC-nanoESI-MS/MS). Mass spectrometry analysis were performed on an Orbitrap Elite instrument (Thermo Scientific) with nanoESI source interfaced to a nanoLC system (Ultimate 3000 RSLCnano System, Thermo Scientific).

The LC/MS–MS analysis on the OrbiTrap ELITE mass spectrometer were performed as follows: digested samples were analysed using an Ultimate 3000 nano-RSLC (Thermo Scientific, San Jose California) coupled in line with an LTQ-Orbitrap ELITE mass spectrometer via a nano-electrospray ionization source (Thermo Scientific, San Jose California). Peptide mixtures were loaded on a C18 Acclaim PepMap100 trap-column (75 µm IDx 2cm,3µm, 100 Å, Thermo Fisher Scientific) for 3.5 min at 5 µl/min with 2% acetonitrile (ACN) and 0.1% formic acid (FA) in H2O, and then separated on a C18 Accucore nano-column (75 mm internal diameter (ID)x50 cm,2.6 mm, 150 Å, Thermo Fisher Scientific) with a 120 min linear gradient from 5 to 50% buffer B (A, 0.1% formic acid (FA) in H2O; B, 80% acetonitrile (acetonitrile(ACN)) and 0.08% FA in H2O) followed with 10 min at 99% B. The total duration was set to 150 min at a flow rate of 200 nl/min. The temperature was kept constant at 40°C.

Acetylated peptides were analysed by Top 10-CID-HCD (collision induced dissociation and high-energy collisional dissociation) data-dependent MS. Tandem mass spectra were searched using SEQUEST HT within Proteome Discoverer 1.4, against a non-redundant protein sequence database for H. sapiens containing 27,858 protein sequence entries (Uniprot, release 2014–11). Cysteine residues were considered to be fully carbamidomethylated (+57 Da statically added), methionine considered to be oxidized (+16 Da dynamically added) and lysine considered to be acetylated (+42 Da dynamically added), and two missed cleavages were permitted. For phosphopeptide analysis a Top20 CID method was applied with Multi Stage Activation to enhance phosphorylation detection (additional activation on phosphate neutral loss at 32.66; 49.00, 65.33 and 98.00 m/z). Peptide mass tolerance was set at 7 p.p.m. and 0.5(CID)/0.02(HCD) Da on the precursor and fragment ions, respectively. The minimum peptide length required was six residues. Proteins with at least two peptides were considered identified. The protein identification list was filtered at a false discovery rate below 1%.

Molecular Modeling:

We carried out the normal mode analysis (NMA) on 20 structures for each construction generated by energy minimization (see below), and the results were averaged to smooth over small variations due to the use of single structures used for a NMA. For the initial energy minimization, a random number of steps was chosen and used in an energy-minimization by the steepest-descent method (SD). this generated 20 different initial structures for the rest of the calculation. Each structure was subjected to up to 20,000 steps of minimization using the adapted basis Newton–Raphson (ABNR) algorithm to reach an RMS gradient of 10^{-7} kcal.mol^{-1} or less. This ensures that the structure is at the local minimum of the potential energy surface, a necessary condition for the NMA. A switching function was used for the van der Waals non-bonded interactions, and a shift function with the distance-dependent dielectric, $\varepsilon = 4r$, was used for the electrostatic interactions. An atom-based 15Å cutoff was used.
Normal-mode calculations : The intrinsic dynamics of HsTop2 were characterized by the atomic root-mean-square fluctuations (RMSF), which were calculated from the normal modes for using the equation

$$\langle(\Delta r_i)^2\rangle = k_B T \sum_{k=7}^{3N} y_{i,k}^2 / (M_i \omega_k^2)$$

where $\Delta ri2$ is the time-averaged mean square displacement of atom i, ωk is the frequency of mode k, $y_{i,k}$ is the displacement of atom i under mode k, N is the number of atoms, and M_i is the mass of atom i (63,64). The fluctuations calculated for each form were averaged over the respective 20 energy-minimized structures. The normal-mode analysis was done using the VIBRAN module of the CHARMM program (41). The treatment of the nonbond interactions in the normal-mode calculations was the same as that used for the energy minimization. Force field parameters for the acetylated lysine were obtained from the work of Grauffel et. al. (65).

Supplemental references:

62. Frauenfelder, H., Petsko, G.A. and Tsernoglou, D. Temperature-Dependent X-Ray-Diffraction as a Probe of Protein Structural Dynamics. *Nature*, **280**, 558-563 (1979) .

63. Brooks, B.R., Janezic, D. and Karplus, M. Harmonic-Analysis of Large Systems .1. Methodology. J Comput Chem, 16, 1522-1542 (1995) .

64. Marques, O. and Sanejouand, Y.H. Hinge-bending motion in citrate synthase arising from normal mode calculations. Proteins-Structure Function and Genetics, 23, 557-560 (1995) .

65. Grauffel, C., Stote, R.H. and Dejaegere, A. Force field parameters for the simulation of modified histone tails. J Comput Chem, 31, 2434-2451 (2010) .

Identification of the acetylations of recombinant Top2α

(Experiments CBE762, CBE834, CBE976, CBE922, CBE039)

NGYGAk$_{ac}$LCNIFSTK

K168

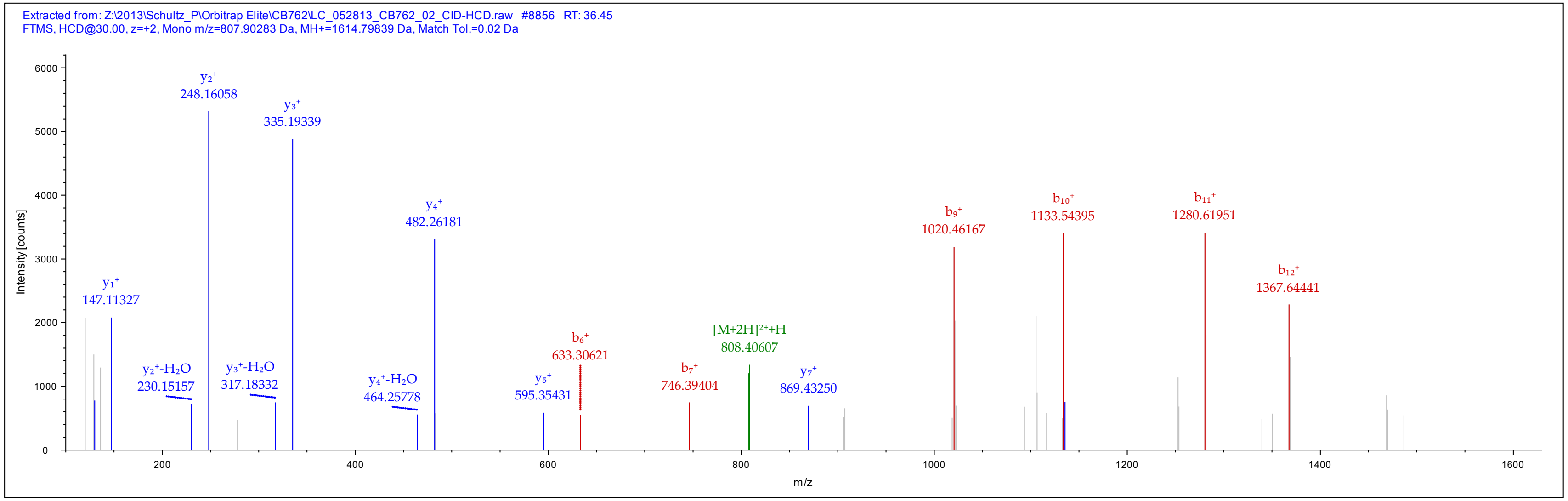

Dk$_{ac}$LDETGNSLK

K278

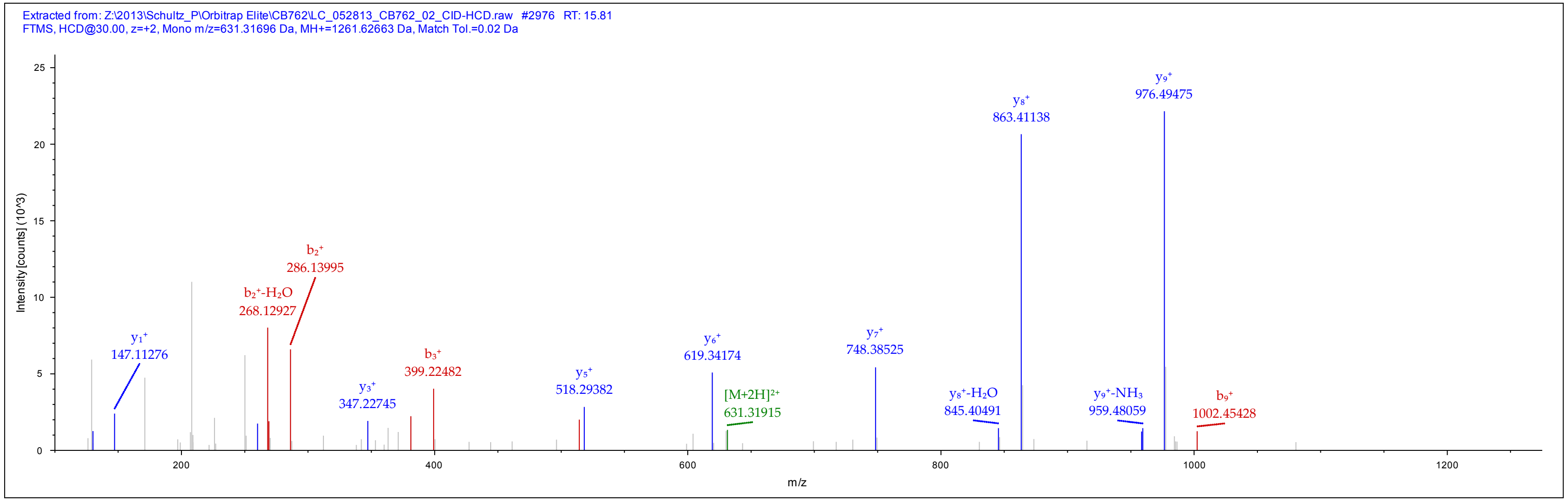

$Dk_{ac}YGVFPLR$

K480

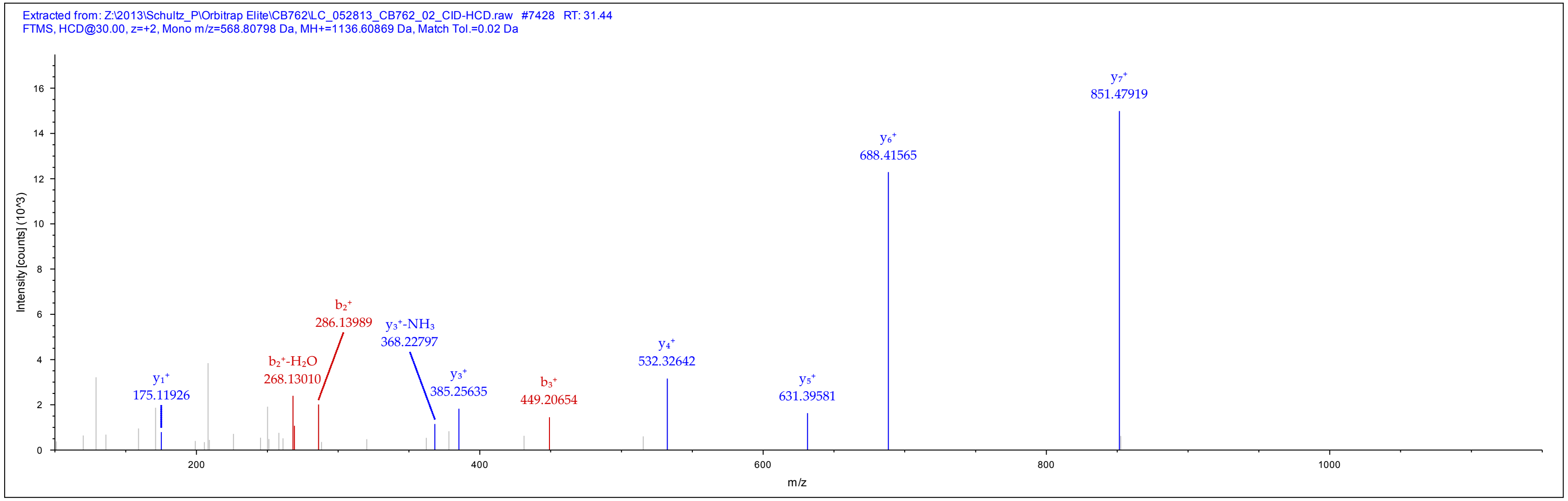

Nk$_{ac}$QEMAFYSLPEFEEWK

K584

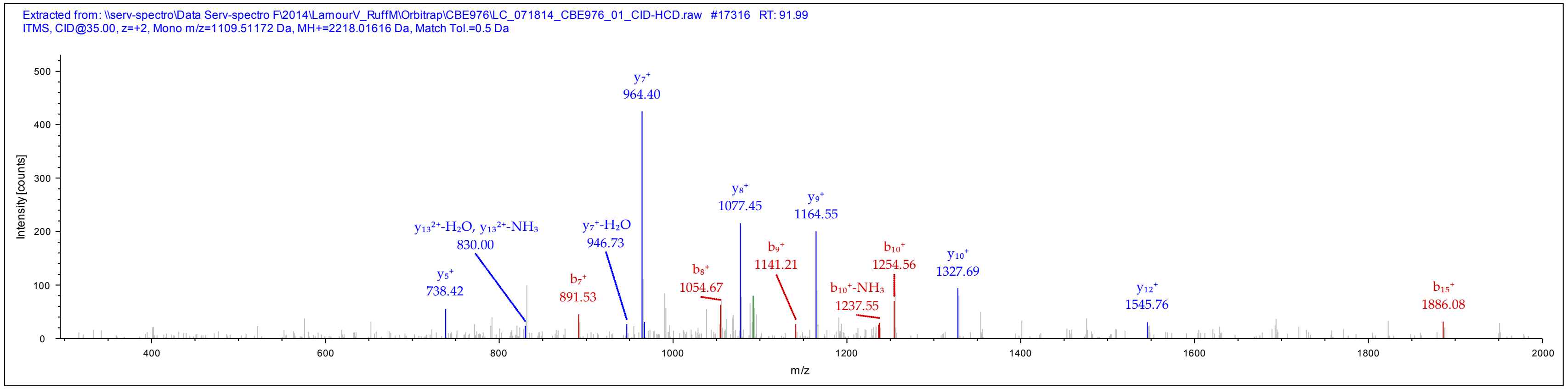

k_{ac}YDTVLDILR

K 1011

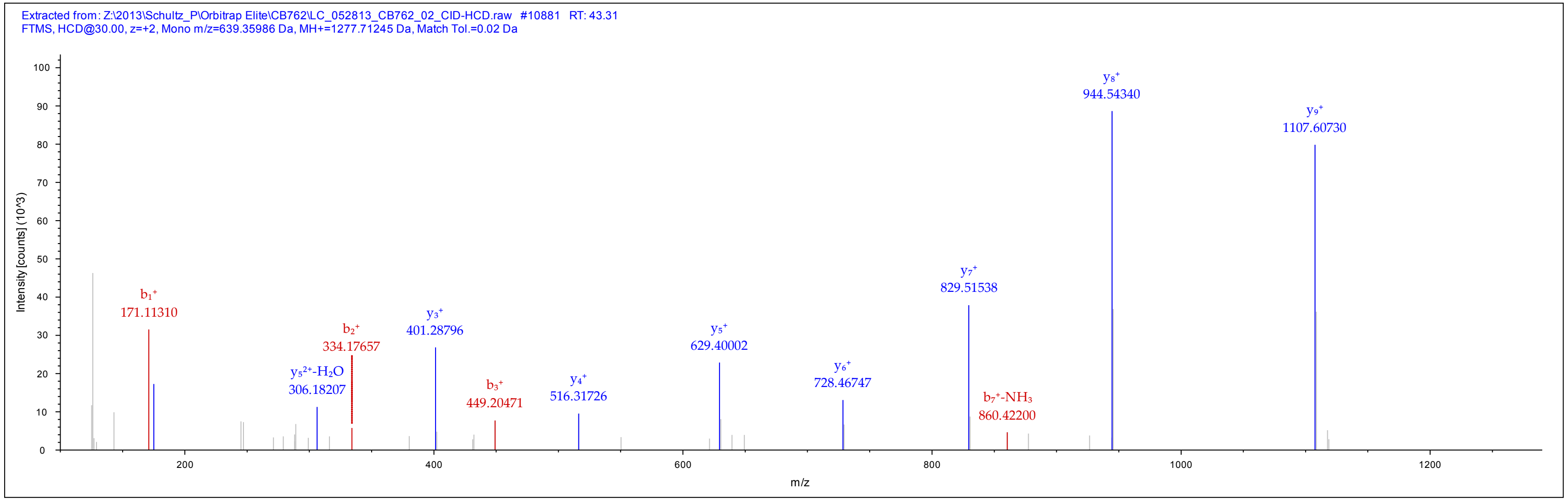

k$_{ac}$EWLLGMLGAESAK

K1034

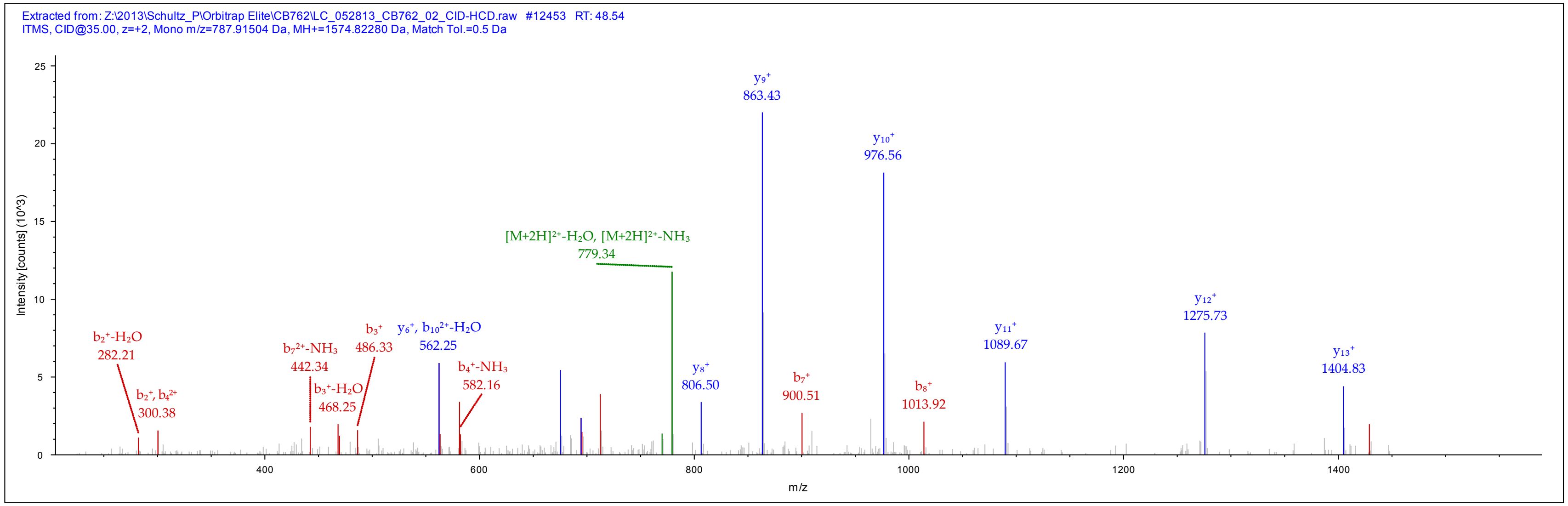

LRNEk$_{ac}$EQELDTLK

K1151

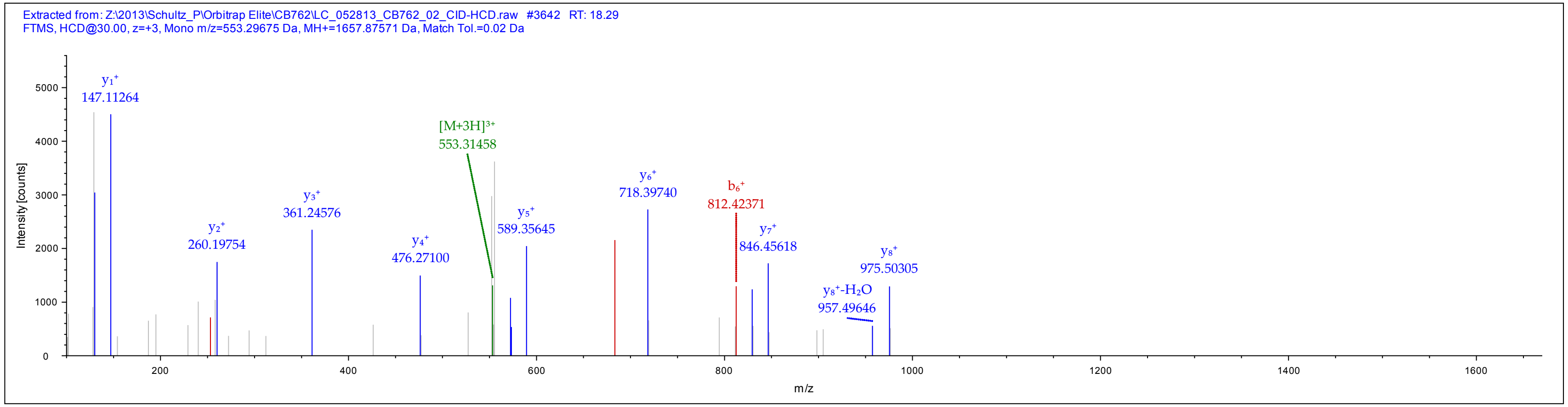

MQSLDk$_{Ac}$DIVALMVR

K233

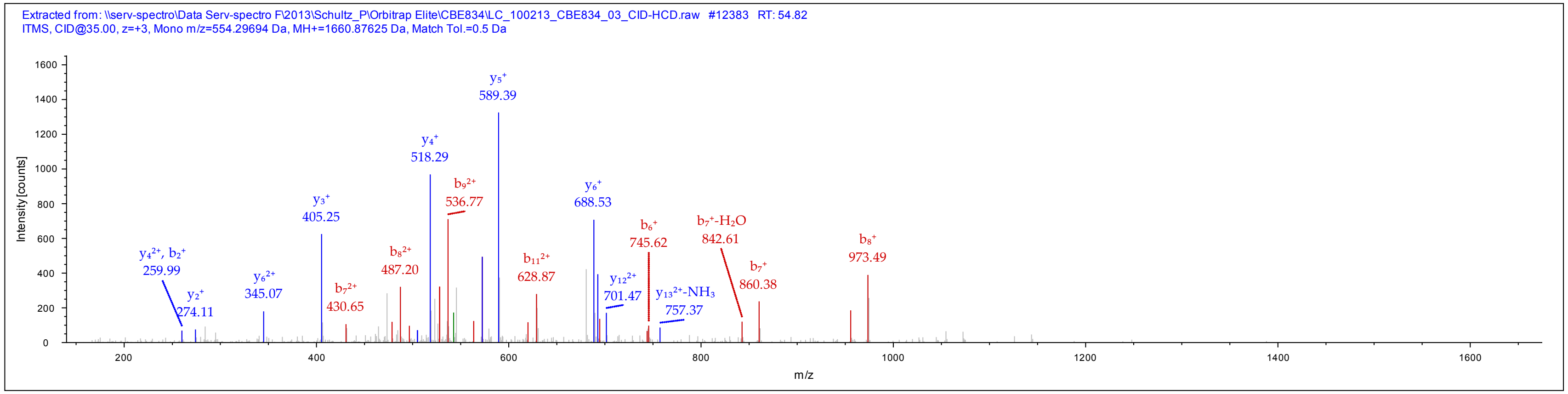

k_{Ac}NYEDEDSLK

K520

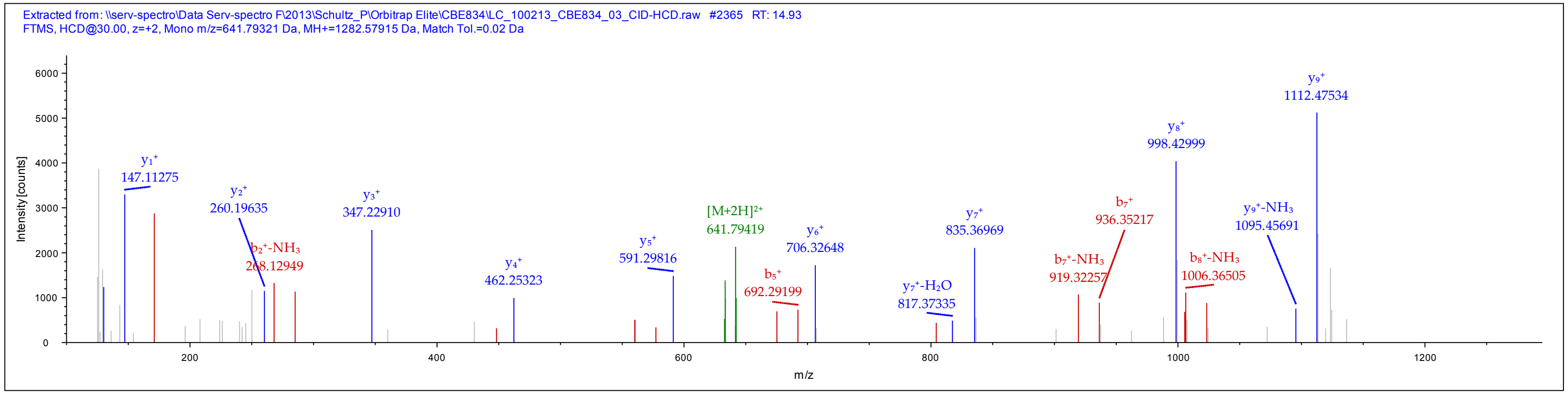

k_{Ac}EWLTNFMEDR

K662

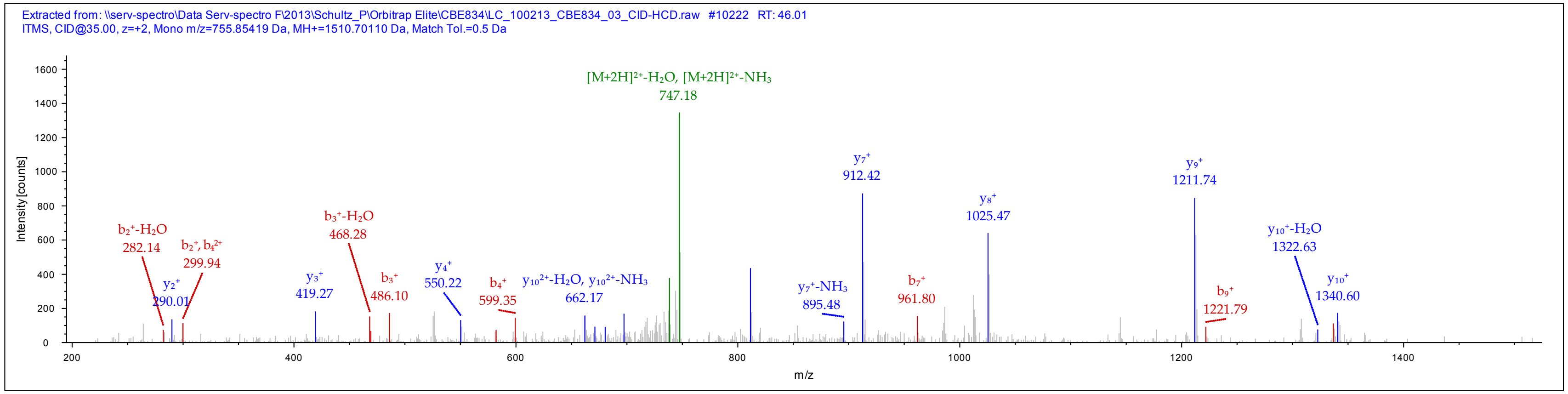

EQVLEPMLNGTEk$_{Ac}$

K949

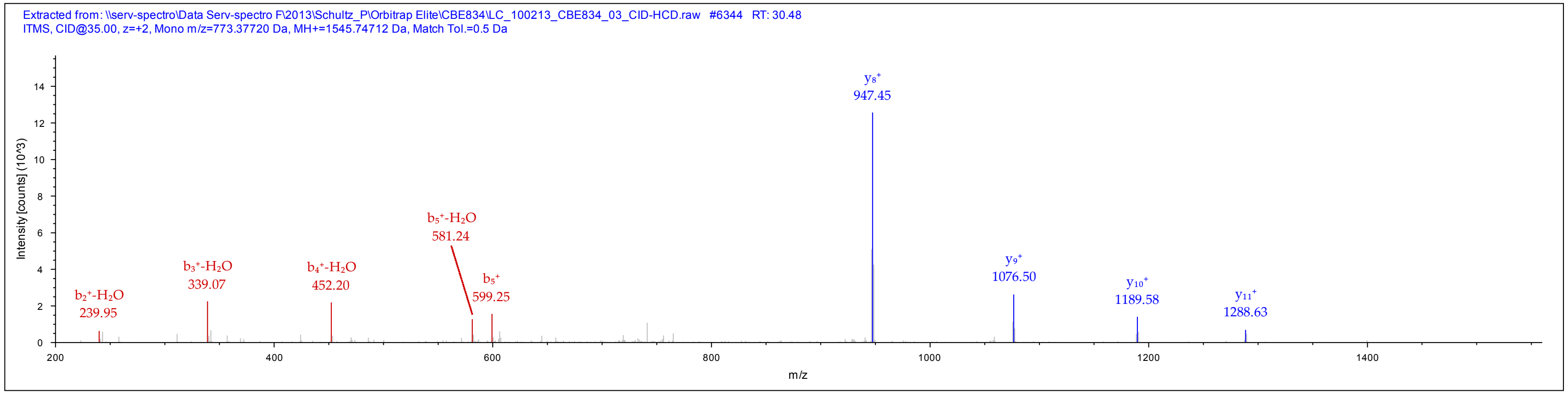

Ik$_{Ac}$NENTEGSPQEDGVELEGLK

K1240

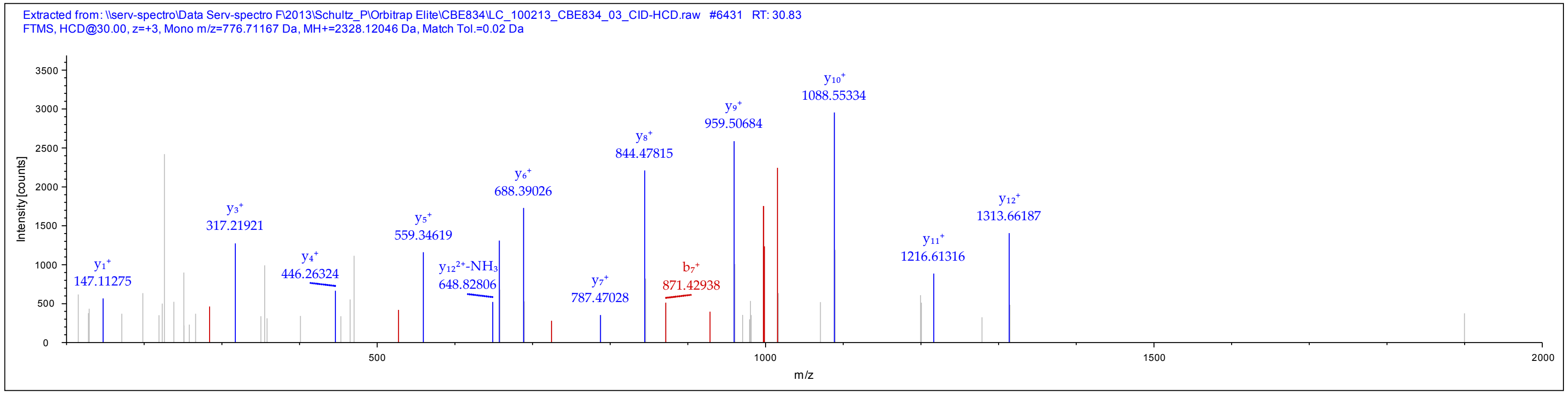

EAQQk$_{ac}$VPDEEENEEs$_{phos}$DNEKETEK

K1096

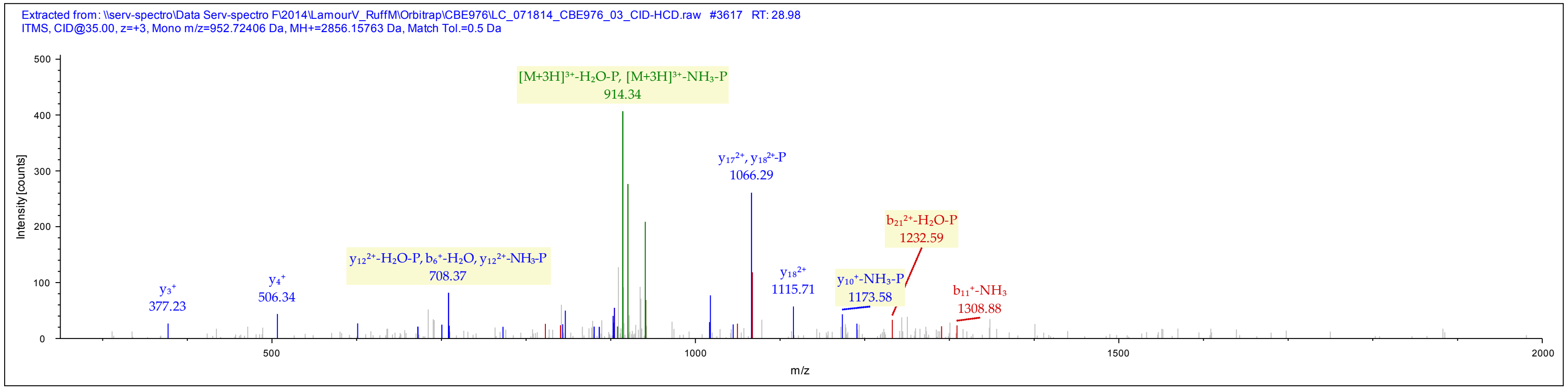

AGEMELk$_{ac}$PFNGEDYTCITFQPDLSK

K207

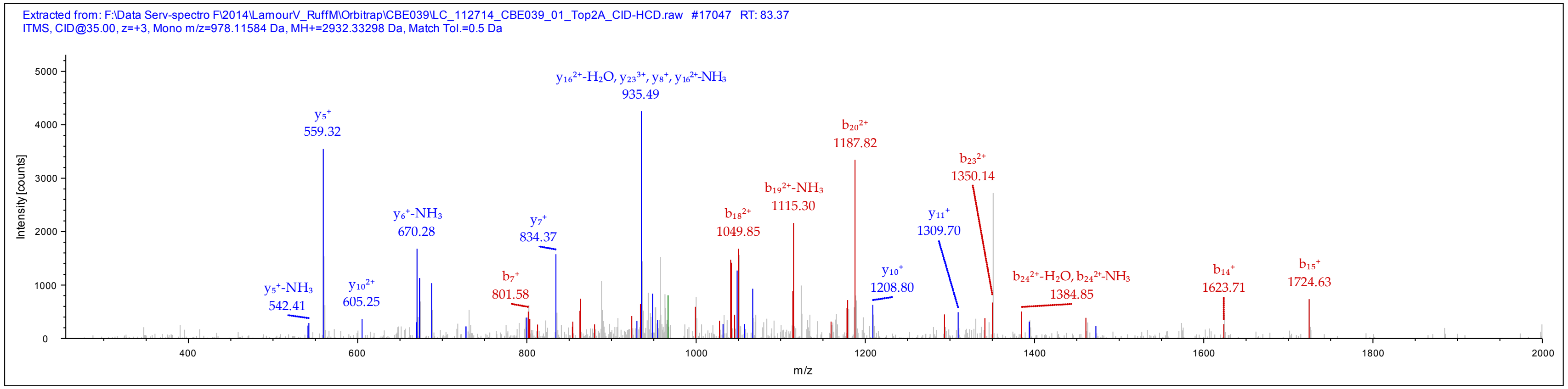

Ek$_{ac}$QDEQVGLPGK

K1186

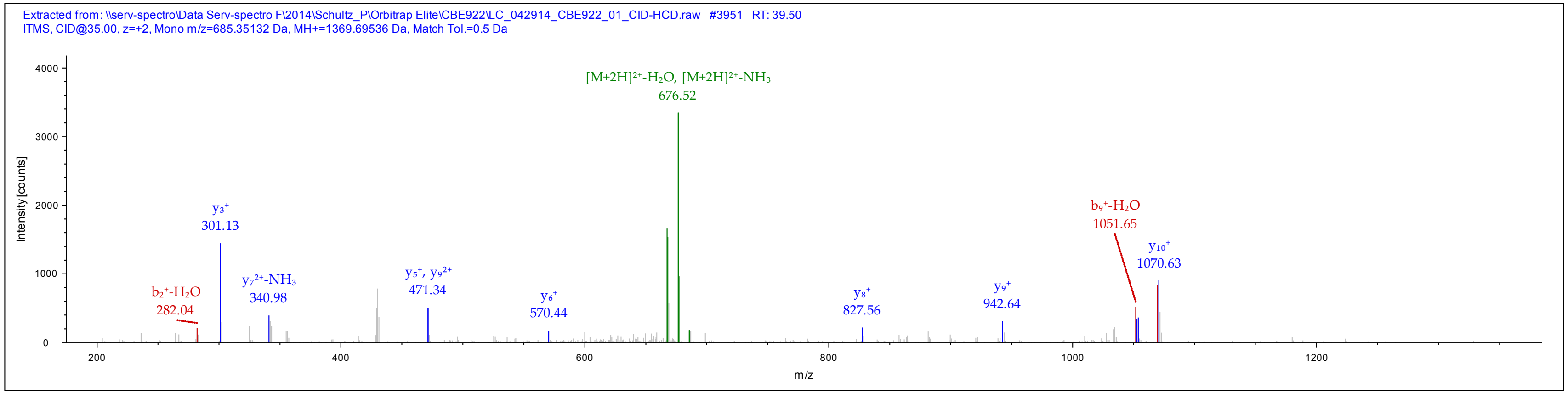

Bedez *et al.*

Raw image Figure S1 (gel picture with protein samples loaded at different concentrations)
Dashed line= displayed area in Figure S1

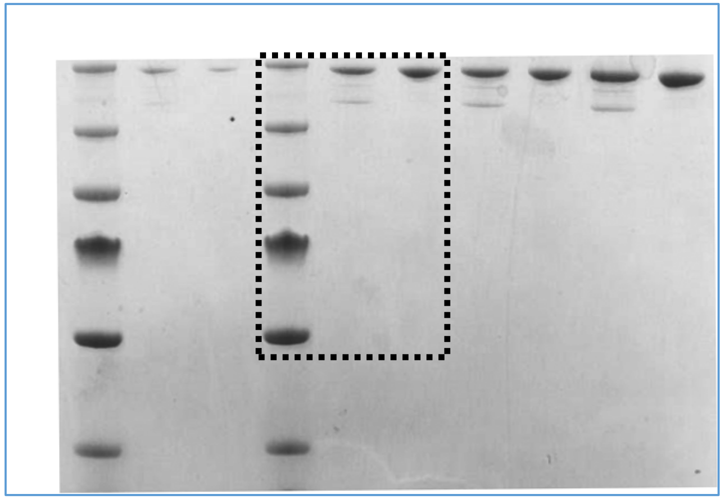

Supplement: Supplementary file 1 — Supplemental information [file 41598_2018_27606_MOESM1_ESM.pdf]
